# A novel methodology to study nanoporous alumina by small-angle neutron scattering

**DOI:** 10.1107/S160057671900726X

**Published:** 2019-06-28

**Authors:** Anastasia Christoulaki, Alexis Chennevière, Isabelle Grillo, Lionel Porcar, Emmanuelle Dubois, Nicolas Jouault

**Affiliations:** a Sorbonne Université, CNRS, Laboratoire Physicochimie des Electrolytes et des Nanosystèmes Interfaciaux, PHENIX, F-75005 Paris, France; b Laboratoire Léon Brillouin (LLB), CEA Saclay, 91191 Gif-Sur-Yvette, France; c Institut Laue–Langevin (ILL), 71 avenue des Martyrs, CS 20156, 38042 GRENOBLE Cedex 9, France

**Keywords:** nanoporous alumina, small-angle neutron scattering, nanochannels, structural characterization

## Abstract

Nanoporous anodic aluminium oxide (AAO) membranes are promising host systems for confinement of condensed matter. Characterizing their structure and composition is thus of primary importance for studying the behavior of confined objects. Here a novel methodology to extract quantitative information on the structure and composition of well defined AAO membranes by combining small-angle neutron scattering (SANS) measurements and scanning electron microscopy (SEM) imaging is reported. In particular, (i) information about the pore hexagonal arrangement is extracted from SEM analysis, (ii) the best SANS experimental conditions to perform reliable measurements are determined and (iii) a detailed fitting method is proposed, in which the probed length in the fitting model is a critical parameter related to the longitudinal pore ordering. Finally, to validate this strategy, it is applied to characterize AAOs prepared under different conditions and it is shown that the experimental SANS data can be fully reproduced by a core/shell model, indicating the existence of a contaminated shell. This original approach, based on a detailed and complete description of the SANS data, can be applied to a variety of confining media and will allow the further investigation of condensed matter under confinement.

## Abstract 2

This work reports a novel methodology to extract quantitative information from small-angle neutron scattering measurements of nanoporous alumina.

## 1. Introduction   

The confinement of condensed matter in nanoporous media can induce at the nanoscale drastic structural or dynamical changes that ultimately lead to original properties that are not achievable under bulk conditions (Huber, 2015 ▸). Such behavior can be then exploited to design new devices or materials with enhanced properties (Md Jani *et al.*, 2013 ▸). Among various nanoporous materials that can be used as a confining medium (clays, mesoporous silica, Vycor glass, nanotubes…), nanoporous anodic aluminium oxide (AAO) membranes are very interesting host systems for confinement studies (Shin *et al.*, 2007 ▸; Krutyeva *et al.*, 2013 ▸). AAOs are obtained through the controlled anodization of aluminium in acidic electrolytes (Masuda & Fukuda, 1995 ▸) and are composed of high-aspect-ratio parallel cylindrical channels with perfectly tunable pore diameters (*D*_p_ from 10 to 100 nm), interpore distances (*D*_int_ from 20 to 200 nm), lengths (*L*_p_ up to 500 µm) and porosities. AAO nanoporous materials are frequently used thanks to their numerous applications in which the control of structural parameters and composition is important: biosensing (Xiao *et al.*, 2016 ▸), templating for the growth of functional materials (Lee & Park, 2014 ▸; Sousa *et al.*, 2014 ▸), and filtration or chemical separation devices (Mozalev *et al.*, 2001 ▸).

A still challenging issue is to find experimental strategies to fully describe the confining medium. The pore-size distribution and porosity of porous materials can be investigated on scales from nanometres to hundreds of micrometres by different methods (Anovitz & Cole, 2015 ▸). Most of the previous work done on AAOs (Le Coz *et al.*, 2010 ▸; Napolskii *et al.*, 2012 ▸; Lee & Park, 2014 ▸) used direct imaging techniques [scanning or transmission electron microscopy (SEM or TEM) and atomic force microscopy] coupled with energy dispersive X-ray (EDX) spectroscopy to characterize the AAOs. These techniques provide information about the pore surface organization and composition as well as section views of nanochannels. However, they have some limitations, such as the limited area studied and the impossibility of having access to the composition inside the whole sample.

Combining these direct imaging methods with scattering techniques can overcome these issues by giving access to averaged structural information over a larger volume (Gommes *et al.*, 2016 ▸) and can also provide information about the pore orientation in both transverse and longitudinal directions. X-rays (via scattering or diffraction) have been mainly used to characterize the pore arrangement and long-range ordering (Dore *et al.*, 2002 ▸; Engel *et al.*, 2009 ▸; Napolskii *et al.*, 2010 ▸; Waheed *et al.*, 2011 ▸; Roslyakov *et al.*, 2013 ▸, 2016 ▸) by tilting the sample along the vertical or horizontal axis normal to the beam. In particular, the different longitudinal pore orientations can be extracted (Roslyakov *et al.*, 2013 ▸), and a strong correlation between transverse and longitudinal pore ordering has been observed (Roslyakov *et al.*, 2016 ▸).

However, the internal composition is still not available. To that end, small-angle neutron scattering (SANS) appears to be a powerful tool to characterize both the pore organization and the chemical composition by determining the different scattering length densities (SLDs) of the material using the contrast variation method (Marchal & Demé, 2003 ▸; Lagrené & Zanotti, 2008 ▸; Grigoriev *et al.*, 2010 ▸; Lefort *et al.*, 2011 ▸). This method consists of canceling the confining medium scattering using an appropriate mixture of deuterated/hydrogenated solvents whose SLD matches that of the confining medium, known as the ‘matching point’. Such an approach allows the determination of the composition of the material. However previous work done on AAO membranes showed that AAOs cannot be perfectly matched (Marchal & Demé, 2003 ▸; Lagrené & Zanotti, 2008 ▸). The contrast variation shows a minimum but the scattering intensities remain high. Imperfect matching comes from inhomogeneities in composition in the AAO due to the presence of contaminants (anions from the electrolyte or water molecules) that organize as a shell around the pores (Mata-Zamora & Saniger, 2005 ▸; Le Coz *et al.*, 2010 ▸; Han *et al.*, 2013 ▸). Perfect matching is thus not experimentally achievable and the scattering signal becomes more difficult to analyze. So far, owing to such complexities, few studies have attempted to fully reproduce the experimental scattering data. Marchal *et al.* (2001 ▸) fitted the SANS high-*q* range of commercial 60 µm-thick anodic membranes by a Porod law to extract their specific surface. Lefort *et al.* (2011 ▸) used a combination of a cylinder form factor with a hexagonal structure factor to fit a homemade AAO under one contrast condition, considering that AAOs are homogeneous in composition. The only attempt to fit the data with a core/shell model was made by Engel *et al.* (2009 ▸) on AAOs measured in air by SAXS, but they did not discuss the obtained SLD values.

Here one has to mention the multiple scattering (MS) effects. MS is the probability for a neutron to be scattered several times and specifically arises in nanoporous materials because of the great length and number of nanopores (Marchal & Demé, 2003 ▸; Grigoriev *et al.*, 2010 ▸; Turkevych *et al.*, 2012 ▸). MS creates an additional *q*-dependent contribution to the total scattering which progressively becomes dominant compared with the AAO scattering. Although some data correction strategies have been proposed in a few studies dealing with weak MS (Goyal *et al.*, 1983 ▸), MS is generally undesirable because this high extra contribution cannot be treated in the context of the single scattering approximation and analytically corrected (Grillo, 2008 ▸). As a consequence the fitting of the scattering curve is mostly impossible.

In this context, this paper aims to describe a detailed strategy to extract quantitative information about the AAO structure and composition. We determine the best experimental conditions to avoid MS and discuss a fitting method, combining SEM analysis and SANS measurements, that leads to a detailed and complete description of the SANS data. This original approach, which can be applied to a variety of confining media, is a required step to further investigate the behavior of condensed matter under confinement.

## 2. Experimental methods   

### 2.1. AAO synthesis   

AAO was prepared using the classical two-step anodization (Masuda & Fukuda, 1995 ▸) of pure aluminium. High-purity aluminium foil (Al, 0.32 mm thick, 99.999% from Goodfellow) was rinsed with acetone and electropolished in a solution of ethanol/perchloric acid (75:25 *v*/*v*) under 15 V for 30 s. Then a first anodization was carried out over a period of 2 h in 0.3 *M* oxalic acid (OA, anhydrous from Alfa Aesar) at different temperatures (4, 10, 18 or 22°C) under a constant voltage of 40 V. After this first anodization, the formed oxide was removed in phosphochromic acid (6 wt% H_3_PO_4_ and 1.8 wt% CrO_3_) at 60°C over a period of 2 h 30 min, the Al substrate keeping the imprints of the nanopores. A second-step anodization was then performed under the same conditions, leading to the formation of self-ordered AAOs. Note that alumina grows normal to the Al foil, producing an equivalent AAO thickness on both sides of the foil. The total AAO thicknesses, which take into account both sides, were determined by SEM (see Table 1 ▸) and can be tuned by varying the anodization temperature and time. The temperature affects the current density *j* and thus the AAO growth rate, and, for a given temperature, a longer anodization time produces thicker AAOs. Table 1 ▸ presents the different experimental conditions used in this study. We choose as sample name nomenclature OA-temperature (in °C)-time (in h).

### 2.2. Scanning electron microscopy   

Information about the nanochannel length, pore size and organization was obtained by SEM imaging performed on a field emission gun scanning electron microscope (FEGSEM, SU-70 Hitachi). Observations were carried out in low-voltage and low-current conditions in order to characterize the insulating sample surface without any coating. Several accelerating voltages were tested, and the best image quality was obtained between acceleration voltages of 1 and 3 kV, while it was not possible to acquire images at lower and higher voltages because of high noise and charging effects, respectively. The pore diameters were determined using the 3 kV images, because they provided deeper beam penetration and better brightness. Images with different magnifications (×10 000, ×20 000, ×50 000 and ×100 000) were recorded to provide information about the long-range pore arrangement and a good pore-size resolution.

EDX spectroscopy measurements were also performed on the samples for elemental surface determination. The spectrometer used was an OXFORD X-Max SDD (crystal 50 mm^2^). The device was mounted on the FEGSEM. The software was an INCA version using XPP modeling for spectrum analysis. Analyses were performed at 5 kV on uncoated specimens and no charging effect was observed. Standard references were used for quantification.

### 2.3. Small-angle neutron scattering   

The pore organization and the AAO composition can be probed by SANS from the analysis of the scattering intensity and the determination of the AAO SLD. For aligned anisotropic objects with low size dispersity the scattering intensity *I*(*q*) [with the magnitude of the scattering vector *q* defined as *q* = (4π/λ)sinθ, where θ is half the scattering angle and λ is the wavelength of the incident neutrons] in the local monodisperse approximation (Pedersen, 1994 ▸) is generally expressed by the relation (1):
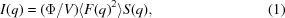
with Φ the volume fraction of scattering objects (here nanopores), *V* = π(*D*_p_/2)^2^ *L*_p_ the volume of the nanopore, 〈*F*(*q*)〉 the averaged scattering amplitude of the object form and *S*(*q*) the structure factor. Our strategy to fit the *I*(*q*) of AAOs will be detailed below.

SANS measurements were performed on the PAXY spectrometer at Laboratoire Léon Brillouin (CEA Saclay, France) and the D11 spectrometer at Institut Laue–Langevin (ILL, Grenoble, France). On PAXY, three configurations were used: 6.7 m/15 Å, 3 m/6 Å and 1 m/6 Å, covering a *q* range from 2 × 10^−3^ to 0.5 Å^−1^. Data reduction was performed with the in-house *PASINET* software using the standard procedure (Brûlet *et al.*, 2007 ▸), in which the sample thickness (which corresponds here to the pore channel length) and transmission (Tr) are required. On D11, two runs were performed (Jouault *et al.*, 2016 ▸; Chennevière *et al.*, 2018 ▸) and four configurations were used: 39 m/6 Å, 16 m/6 Å, 8 m/6 Å and 1.4 m/6 Å, covering a *q* range from 2 × 10^−3^ to 0.4 Å^−1^. Data reduction was performed using the ILL *GRASP* or *Lamp* software (Dewhurst, 2018 ▸; Richard *et al.*, 1996 ▸).

Membranes of around 1 × 1 cm with AAO present on both sides of the Al foil were measured either in air or in H_2_O/D_2_O mixtures. For measurements in solvent, the AAOs were immersed in the mixtures for some seconds to be wetted and to avoid the presence of air bubbles, and then placed in circular cells between quartz windows separated by a 300 µm Teflon o-ring corresponding to the total sample thickness. The cells were then aligned to have the pore channels oriented parallel to the neutron beam. The pore alignment was done by evaluating the isotropy of the 2D scattering pattern on the detector. Ideally, such patterns consist of concentric rings for a perfectly aligned sample. To precisely achieve this, the following procedure, depicted in Fig. S1 in the supporting information, was used. The sample was tilted in one direction (*X* or *Y*) to create strong anisotropy and the appearance of non-aligned spots in the other direction (*Y* or *X*). The alignment of the spots in this direction was then ensured by rotating the sample stepwise using a goniometer. This operation was repeated in the other direction, and the final alignment was inspected by performing a fast measurement to check the pattern isotropy. The spectra were then treated as isotropic using the whole detector.

Finally, since the AAO coherent scattering becomes negligible at high *q*, the incoherent background was subtracted by removing a constant value corresponding to the flat incoherent signal from the solvent, *i.e.* the constant value at high *q*. Note that measurement of AAO in air showed that the incoherent signal from the AAO is very low [around 10^−3^ cm^−1^, see Fig. 2(*b*)].

## 3. Results and discussion   

In the following the detailed strategy to model the SANS data will be described step by step. This approach is based on the combination of SEM analysis to extract the structure factor *S*(*q*) and SANS experiments performed in optimal conditions to extract quantitative parameters on the nanostructure and composition.

### 3.1. Determination of the structure factor *S*(*q*) by SEM analysis   

**Figure 1:**
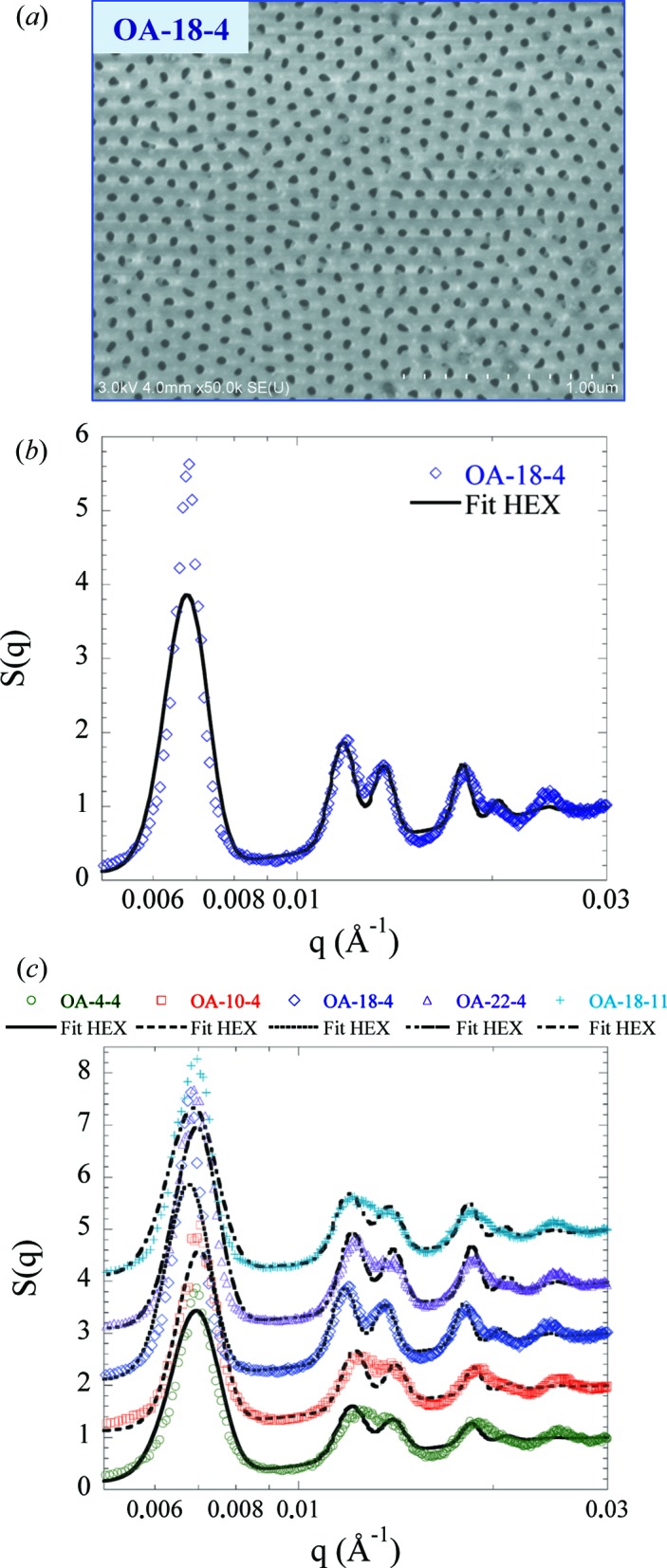
(*a*) SEM top surface image of OA-18-4. (*b*) Structure factor *S*(*q*) derived from SEM-image FFT for OA-18-4 (blue circles). The continuous black line corresponds to the best fit using a hexagonal model. (*c*) *S*(*q*) of all AAO samples. From bottom to top: OA-4-4, OA-10-4, OA-18-4, OA-22-4 and OA-18-11. The curves have been vertically shifted for clarity.

As shown by relation (1), *S*(*q*) contributes to the SANS scattering intensity and can be independently determined by SEM image analysis. Fig. 1 ▸(*a*) shows a SEM image of the top surface of AAO membrane OA-18-4. The fully open nanopores are hexagonally ordered in domains of average lateral size *D* with different orientations. From SEM analysis (*i.e.* image binarization followed by a pore-size distribution analysis), we find a pore diameter (*D*_p_) of 42.0 ± 3.8 nm (relative pore distribution σ_p_ = 0.09), a porosity (*P*) of 14% and a pore density of 1.0 × 10^10^ pores cm^−2^. Similar analyses were performed on all AAO samples (SEM images are shown in Fig. S2), and Table 2 ▸ summarizes all the different characteristic sizes. First, the anodization time affects the pore diameter and porosity as the sample is gradually dissolved with time by the acidic electrolyte (OA-18-4 versus OA-18-11). Then, for a given anodization time, *D*_p_ and *P* increase with temperature while the pore density remains almost unchanged, as already observed in the literature (Lee & Park, 2014 ▸).

To go further and quantify the pore arrangement and ordering in the transverse direction, the 2D fast Fourier transform (FFT) of the pore-center positions from SEM images at low magnification (×10 000) was performed to compute the structure factor *S*(*q*) [see Fig. 1 ▸(*b*) for OA-18-4]. *S*(*q*) presents different peaks whose positions are directly related to the pore ordering. Depending on the degree of pore ordering, *S*(*q*) can be reproduced by either a hard-disk model for less ordered AAO (Rosenfeld, 1990 ▸; Engel *et al.*, 2009 ▸) or a hexagonal model for clearly ordered AAO (Engel *et al.*, 2009 ▸). Here *S*(*q*) shows characteristic peaks of a hexagonal lattice and can be fitted assuming a hexagonal pore arrangement with a lattice parameter *a* (*i.e.* the interpore distance *D*_int_) expressed as follows (Förster *et al.*, 2005 ▸):
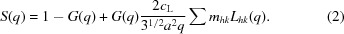

*G*(*q*) is the Debye–Waller factor, which is a disorder parameter taking into account the distortion of the lattice through the relative interpore distance distribution σ_*a*_:
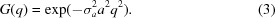
And 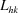
(*q*) is the normalized peak shape Gaussian function:
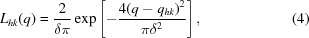
with δ the peak width related to the domain size *D* as *D* = 2π/δ. In relation (2), 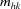 is the multiplicity factor (here fixed to 3) and *c*_L_ is a correction factor for the Porod invariant, which will be a free parameter in our fitting strategy (Sundblom *et al.*, 2009 ▸).

Equation (2) fits the experimental *S*(*q*) of OA-18-4 as seen by the continuous line in Fig. 1 ▸(*b*) with *D*_int_ = 106.4 nm, σ_*a*_ = 0.063 and δ = 8.2 × 10^−4^ Å^−1^, giving a domain size *D* of 766 nm. Note that the amplitude of the first peak is not perfectly reproduced by the model. It can be better adjusted (curve not shown) by decreasing δ to 6 × 10^−4^ Å^−1^, *i.e.* *D* = 1047 nm, and increasing σ_*a*_ to 0.072. However, using these parameters, the adequacy of the model for the other peaks is lost. This can be explained by the fact that the experimental *S*(*q*) contains information about the polydispersity of transverse pore positions and also about the polydispersity of the transverse orientations of ordered domains. Thus we chose to reproduce all the peaks to have a better picture of the pore ordering.

All the produced AAOs can be fitted with the hexagonal model as shown in Fig. 1 ▸(*c*). The different fitting parameters are listed in Table 2 ▸. The AAOs all adopt a hexagonal organization with similar interpore distance (which is fixed by the voltage during anodization) and domain size, in good agreement with previous work, showing that temperature has a weak influence on ordering while electrolyte concentration and applied voltage can modify the pore arrangement (Ba & Li, 2000 ▸). Thanks to SEM analysis *S*(*q*) can be determined and used during the SANS fitting.

### 3.2. Canceling of multiple scattering   

SANS is a powerful technique to characterize both the pore organization and the chemical composition by determining the different SLDs of the material through a fit of the experimental data. However, as mentioned in the *Introduction* [Sec sec1], careful measurements are required prior to any fits of the SANS data to provide suitable conditions for data interpretation.

**Figure 2:**
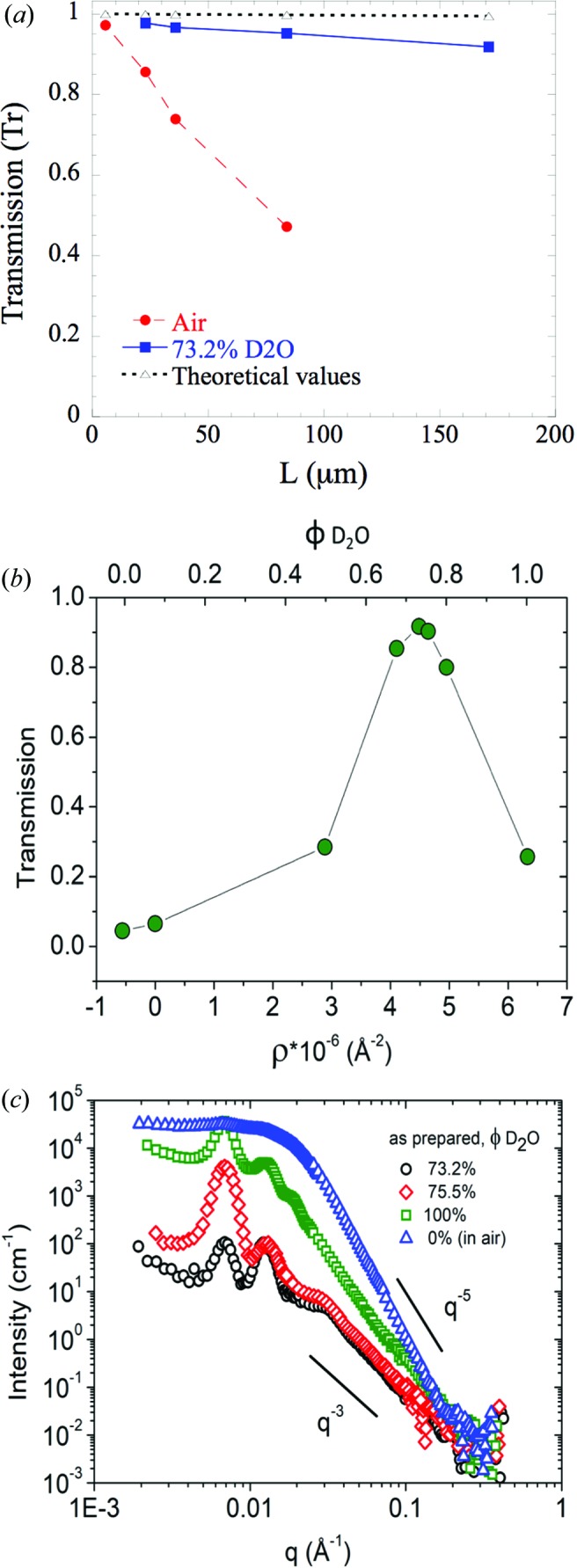
(*a*) Transmission evolution as a function of AAO length in air (red circles) and in 73.2% D_2_O solvent (blue squares). The theoretical Tr values are plotted as black triangles. (*b*) Transmission variation with SLD (denoted as ρ). (*c*) Scattering intensities of AAO OA-18-11 for different D_2_O volume fractions: 0% (blue triangles), 73.2% (black circles), 75.5% (red diamonds), 100% (green squares).

In particular, experimental conditions have to be chosen to cancel out MS, because it has a strong influence on the scattering intensity *I*(*q*). A simple approach to detect MS is to measure the neutron transmission (Tr = *N*_tr_/*N*_0_, with *N*_tr_ the number of neutrons transmitted through the AAO and *N*_0_ the number of neutrons of the incident beam) of the AAO (Cousin, 2015 ▸). Tr is defined as Tr = exp(−*n*σ_tot_ *L*), with *n* the number density [= (ρ/*M*)*N*_a_, ρ being the mass density, *M* the molar mass and *N*_a_ the Avogadro number], σ_tot_ = 17.25 barns the absorption cross section of alumina and *L* the AAO length. Thus, the expected Tr for pure Al_2_O_3_ varies from 0.9998 to 0.9948 for the studied length range [see Fig. 2 ▸(*a*)].

Fig. 2 ▸(*a*) shows the Tr evolution of AAOs measured in air (red circles) as a function of AAO length. One can observe a substantial decrease in Tr with *L* compared with the theoretical values. Since the adsorption cross section of alumina is low, such deviations come from MS. Only the very thin AAO membrane (below 6 µm) synthesized for this measurement has a high Tr in air, but such a thickness is in practice very difficult to handle. Another approach to avoid MS is to work with solvents having SLDs close to that of the AAO, *i.e.* close to the AAO matching point. Fig. 2 ▸(*b*) shows the Tr variation with the SLD of the solvent (*i.e.* the D_2_O volume fraction) for OA-18-11. A maximum in Tr of 0.917 is obtained for 73.2% D_2_O, corresponding to an SLD of 4.53 × 10^−6^ Å^−2^. When we move away from this D_2_O proportion, Tr strongly decreases to reach 0.043 in pure H_2_O (SLD of −0.56 × 10^−6^ Å^−2^). Few contrast conditions have Tr close to 0.9, indicating that there is a small contrast range barely affected by MS. Therefore, in the following, we chose to work in 73.2% D_2_O to minimize the MS effects whatever the AAO thickness, as shown in Fig. 2 ▸(*a*) (blue squares).

Now we propose to look at the scattering intensities *I*(*q*) under several contrasts. Fig. 2 ▸(*c*) shows the *I*(*q*) of OA-18-11 (*L*_p_ = 171 µm) immersed in different H_2_O/D_2_O mixtures (0, *i.e.* in air, 73.2, 75.5 and 100% D_2_O). For 73.2% D_2_O, where Tr is maximum and thus MS absent, the SANS signal is characteristic of aligned cylinders parallel to the neutron beam with a typical *q* ^−3^ dependence at high *q*. At low *q*, a peak (located at the position q*) is visible and originates from the pore–pore correlations [*i.e.* the signature of *S*(*q*)]. From the position q* the interpore distance *D*_int_ can be calculated. In the intermediate *q* range, different oscillations are observed coming from *S*(*q*) and the form factor 〈*F*(*q*)^2^〉 of the pores. For 75.5% D_2_O, the SANS scattering has the same general behavior at high *q* and the same peak positions but shows an increase of the first peak amplitude, while the second one decreases. At 100% D_2_O, *I*(*q*) in the intermediate *q* range increases significantly compared with that of the 73.2% D_2_O sample, with a *q* ^−5^ scaling and less pronounced peak amplitudes at low *q*. Note that all the peaks remain at the same position whatever the H_2_O/D_2_O mixture. Since the theoretical AAO absorption cross section is low and AAO is well aligned, this increase is due to MS and is consistent with a low Tr. For 0% D_2_O the MS is so pronounced that all peaks disappear and the intensity reaches values larger then 10^4^ cm^−1^. This additional *q*-dependent contribution caused by MS is due to the high anisotropic structure of the AAO. When MS occurs, the first scattering event occurs at an incident wavevector parallel to the pore axis. The beam is then scattered (mostly at the first correlation peak), leading to tilted incident wavevectors for the following scattering events. The Porod regime for tilted cylinders scales as *q* ^−5^, which is in good agreement with the *q* dependency observed experimentally under MS conditions.

Finally, a low Tr and a deviation from the *q* ^−3^ behavior at high *q* are the two criteria that can provide clear evidence of MS. As a consequence, to avoid MS, we describe our fitting strategy on SANS data measured in 73.2% D_2_O.

### 3.3. Full SANS interpretation: fitting strategy   

In the following a detailed strategy will be presented to fully fit the SANS data in 73.2% D_2_O (*i.e.* without the presence of MS) assuming that AAO can be described with a core/shell aligned-cylinder model. Such an approach has not been attempted so far owing to the complexity of obtaining a reliable SANS spectrum as described above.

#### 3.3.1. Evidence of a contamination layer and estimation of its extent   

As experimentally observed, the AAO membranes are not homogeneous in composition. Contamination from electrolyte anions creates compositional heterogeneities in the AAOs. The porous AAO cell is separated into two layers with different composition (Thompson & Wood, 1981 ▸; Ono & Masuko, 1992 ▸). The first layer is closer to the pore channel and consists of alumina which is contaminated with electrolyte roots such as oxalates, sulfates or phosphates depending on the acid used for the synthesis (Thompson & Wood, 1981 ▸; Le Coz *et al.*, 2010 ▸), and the amount and extent of root incorporation increases with the concentration of the electrolyte and is proportional to the applied voltage (Mínguez-Bacho *et al.*, 2015 ▸). The second layer consists of higher-purity alumina and extends to the skeleton of the porous structure.

**Figure 3:**
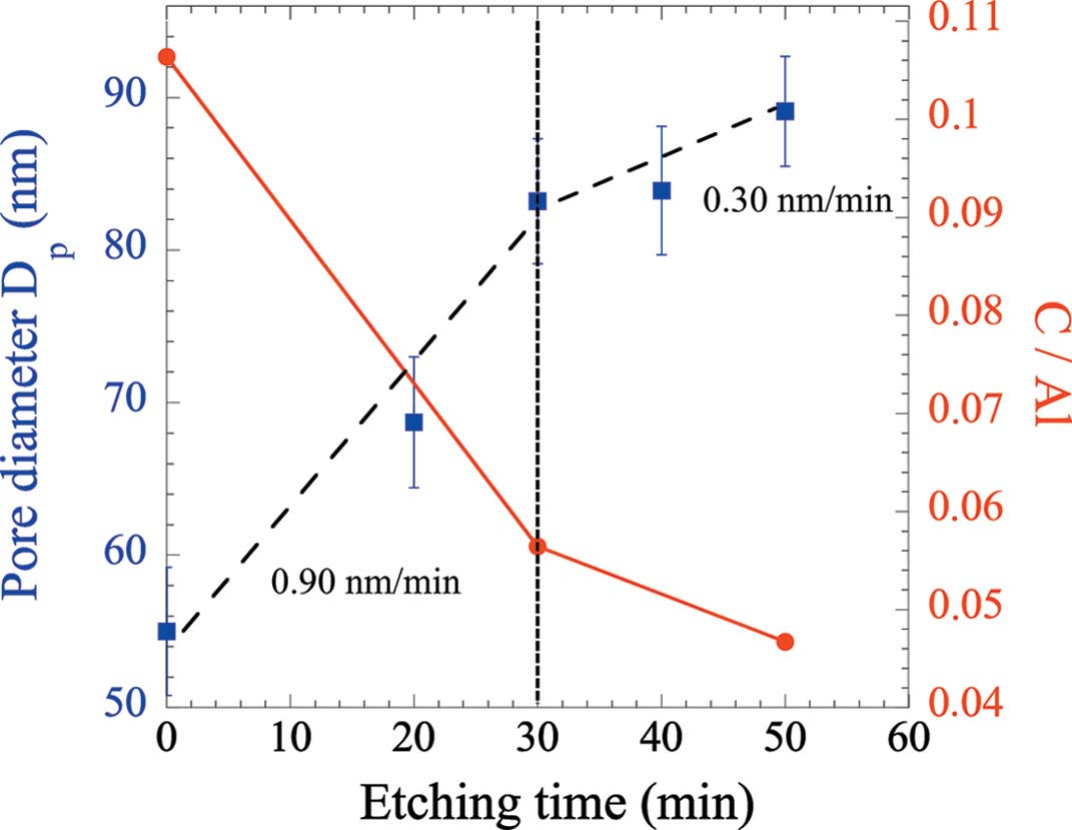
Evolution of pore diameter (blue squares) determined by SEM and C/Al ratio (red circles) determined by EDX with etching time in phosphoric acid (5 wt% at 30°C) of a sample equivalent to OA-18-11.

This contamination (here oxalates, C_2_O_4_^2−^, since we used OA as electrolyte) can be shown by AAO dissolution in phosphoric acid (5 wt% at 30°C) (Han *et al.*, 2007 ▸). Fig. 3 ▸ presents the evolution of the pore diameter *D*_p_ determined by SEM (blue squares) of OA-18-11 as a function of the etching time in acid. Its evolution can reveal changes in the etching dissolution rate which are related to the anion incorporation gradient along the pore wall. It is known that the anion-contaminated oxide has a higher solubility than the rest of the inner layer (Han *et al.*, 2007 ▸; Lee & Park, 2014 ▸). From the *D*_p_ evolution in Fig. 3 ▸, two regimes can be distinguished, and the slopes give the dissolution rates of the different porous alumina regions.

A decrease in the dissolution rate occurs after 30 min (passing from 0.9 to 0.3 nm min^−1^), indicating the crossover between the contaminated layer (high dissolution rate due to the presence of contaminated anions) and the higher-purity alumina (lower dissolution rate). The extent of the anion-incorporated layer can be estimated by the difference between the initial *D*_p_ (*t* = 0) and that at the change of the slope (*t* = 30 min), giving a thickness of about 15 nm for the incorporated layer. This observation is confirmed by further EDX spectroscopy (red circles), showing a decrease of the C/Al ratio as the pore wall gets thinner, the carbon content being directly related to the amount of incorporated oxalate (see the elemental analysis in the supporting information). This experiment showing two distinct compositional regimes indicates that a core/shell cylinder model is suitable for the SANS fitting.

#### 3.3.2. Description of the fitting model   

**Figure 4:**
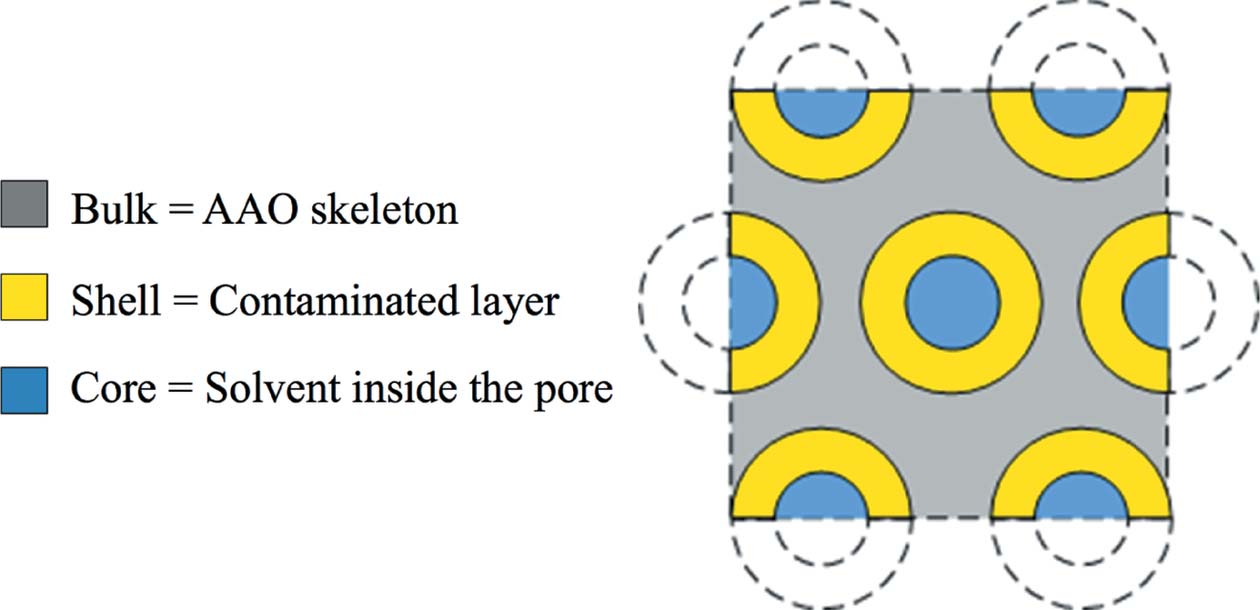
Schematic representation of the core/shell model: the nanopores, corresponding to the core in the fitting model, are filled with solvent (in blue) and the heterogeneity in composition is represented by a shell (in yellow). The remaining skeleton oxide in gray corresponds to the bulk in the model.

The core/shell cylinder model is depicted in Fig. 4 ▸. The core corresponds to the center of the nanopores filled here by the solvent (73.2% D_2_O). The shell corresponds to the contaminated area and the bulk is the purer oxide.

In this model the form factor scattering amplitude for a perfectly aligned cylinder is given by
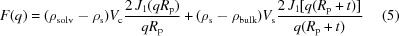
with ρ_solv_, ρ_s_ and ρ_bulk_ the SLDs of the solvent, the shell and the bulk, respectively. *V*_c_ is the volume of the core (*V*_c_ = 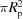 *L*_p_, with *L*_p_ the length of the cylindrical object) and *V*_s_ the volume of the core/shell [*V*_s_ = 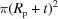 *L*_p_, with *t* the shell thickness]. 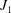 is the first-order Bessel function of the first kind.

The polydispersity in the radius and the shell thickness can also be taken into account with a Gaussian distribution given by the following equation:
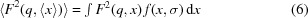
with
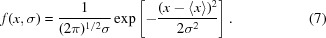
〈*x*〉 is the mean value of either the pore radius *R*_p_ or the shell thickness *t*, and σ is the standard deviation (denoted as σ_p_ or σ_t_ for the radius or shell thickness, respectively). Finally the instrumental resolution was also accounted for through a resolution function *R*(*q*, 〈*q*〉) (Lairez, 1999 ▸) as
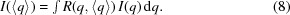



**Figure 5:**
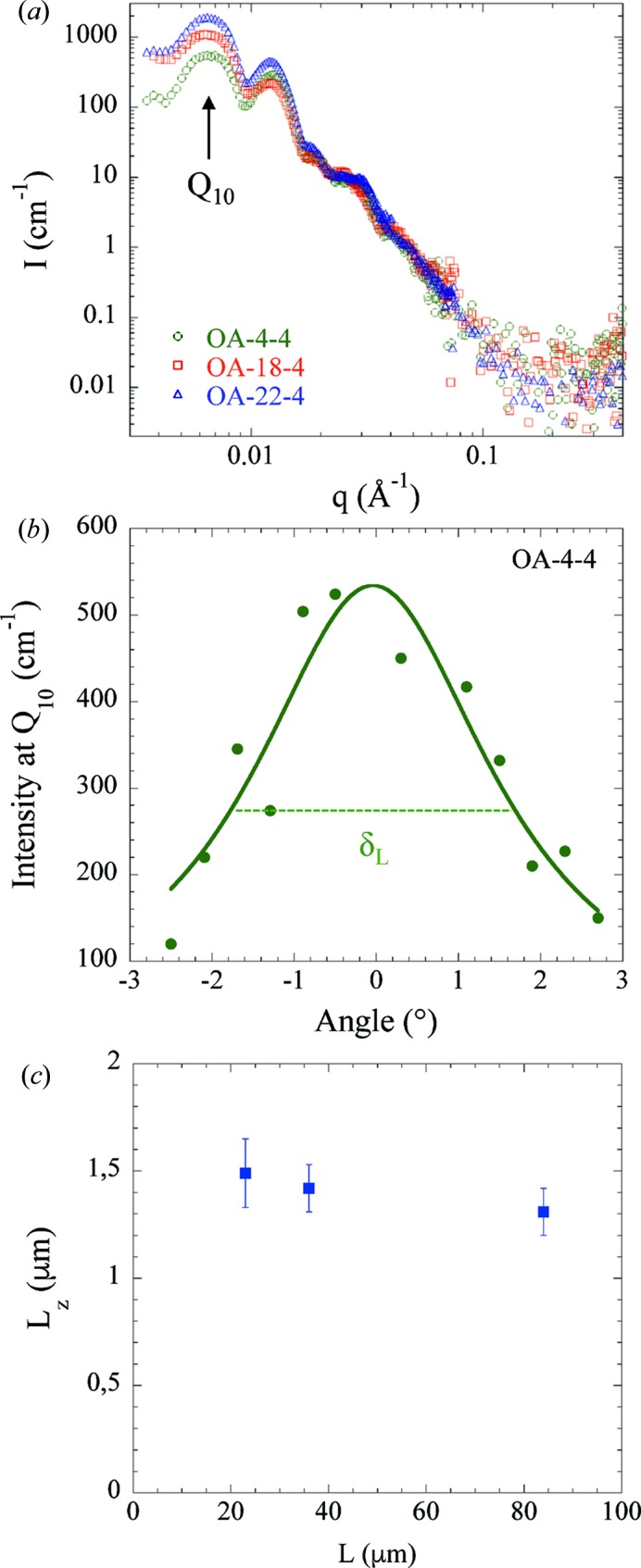
(*a*) SANS scattering intensities *I*(*q*) of OA-4-4, OA-10-4 and OA-22-4, having different thicknesses (*L* = 23, 36 and 84 µm, respectively). (*b*) Rocking curve of OA-4-4 fitted with a Lorentzian distribution (continuous line). (*c*) Longitudinal correlation length *L_z_* as a function of *L*.

In order to avoid any overlapping of the scattering objects, the shell thickness must be smaller than
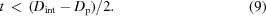
For δ, which quantifies the domain size *D*, we have to consider the coherence length of the neutrons in the transverse direction relative to the beam path, giving the maximum probing length of our sample, *i.e.* the maximum extent in which the neutrons can interfere (Grigoriev *et al.*, 2010 ▸). Here, the value of δ will influence the SANS peak shape in the low-*q* region [see Figs. 5 ▸(*a*) and 6 ▸]. The transverse coherent length of the neutron beam is given by
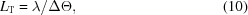
with λ the neutron wavelength and ΔΘ the divergence of the direct beam given by the experimental collimation (Lairez, 2010 ▸). Here, two cases have to be considered. If the domain size *D* of the ordered domains as measured by SEM is smaller than *L*_T_ then the parameter δ will be fixed to that obtained from SEM. If the domain size *D* is larger than *L*_T_ then δ is fixed to the minimal value set by the experimental collimation (see Table S2, which summarizes the different values depending on the instrumental configurations used during our SANS runs). The values of *L*_T_ (>1000 nm) are always larger than *D*, and thus the parameter δ will be fixed to the value determined by SEM analysis.

In the longitudinal direction similar considerations have to be taken into account. Since this length is critical for the SANS fitting, its determination is described in detail in Section 3.3.3.

Finally, the model is composed of many parameters summarized in Table 3 ▸. To reduce the number of fitting parameters we will fix all those extracted from the SEM analysis and will keep as variable *c*_L_, ρ_shell_, ρ_bulk_, *R*_p_ and *t*. Note that *R*_p_ remains a fitting parameter since the value extracted from SEM is generally larger than the actual SANS value because of the chemical etching during the AAO synthesis (Lee & Park, 2014 ▸; Christoulaki *et al.*, 2019 ▸). The data fitting was performed using the *SasView* software (SAS, http://www.sasview.org/) with homemade programs.

#### 3.3.3. Determination of the longitudinal correlation length *L_z_*   

In principle, the fitting of the SANS data in absolute units using the core/shell cylinder model described above can give a full description (structure and composition) of the AAOs. In this model the scattering intensity depends on the length of the AAO [see equation (5)]. However, a particular observation has to be made. Here we will focus on samples OA-4-4, OA-10-4 and OA-22-4 because they have been measured during the same SANS run, *i.e.* they have the same data resolution. Fig. 5 ▸(*a*) shows that the scattering intensities *I*(*q*) of AAOs with different lengths *L* (23, 36 and 84 µm, corresponding to OA-4-4, OA-10-4 and OA-22-4, respectively) superimpose at high *q*, suggesting that the probed length during the SANS experiments is the same whatever the real channel length *L*. Note that the larger differences in *I*(*q*) at low *q* might arise from differences in AAO composition and structure when the anodization temperature is increased to produce thicker AAOs. Grigoriev *et al.* (2007 ▸) proposed that the probed cylinder length to put in the SANS fitting is the longitudinal correlation length *L_z_* of the lattice, *i.e.* the ordering length along the pore channel. *L_z_* is determined by measuring the variation in the scattered intensity of the *Q*_10_ component parallel to the cylinder axis *Q_z_* as a function of the tilting angle of the pore axis with respect to the incident beam. *L_z_* is then defined as
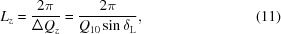
where *Q*_10_ is the 10 correlation peak position and 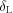 the width at half-maximum of the rocking curve.

It must be emphasized that such a procedure is no longer valid when MS occurs, justifying the use of the 73.2% D_2_O solvent that cancels out MS (see above). Fig. 5 ▸(*b*) shows the rocking curve of OA-4-4 fitted with a Lorentzian distribution, and, using equation (11), a value of *L_z_* = 1.49 µm has been found. *L_z_* corresponds to the ordering distance, or grain size, in the longitudinal direction, *i.e.* along the pore channel. This value is of the same order of magnitude as the transverse size *D* (0.66 µm for OA-4-4) determined previously. Fig. 5 ▸(*c*) shows the different *L_z_* obtained for OA-4-4, OA-10-4 and OA-22-4. *L_z_* appears to be independent of the real length *L* of the AAOs, an observation consistent with the superimposition of *I*(*q*) in Fig. 5 ▸(*a*). Similar analyses have been performed for OA-18-4 and OA-18-11, and *L_z_* values of 1.74 and 1.33 µm, respectively, have been found. These results show that *L_z_* is a critical parameter to fully interpret the SANS data and, in the following fitting, we will use the values of *L_z_* as the length in equation (5).

#### 3.3.4. Application of the fitting model to AAOs   

The described strategy has been used to fit AAOs prepared in oxalic acid under different conditions as presented in Table 1 ▸. In particular, this approach can be used to quantify the effect of anodization conditions (time and temperature) on the AAO structure and composition by following both the form factor 〈*F* ^2^(*q*)〉 and the structure factor *S*(*q*).

**Figure 6:**
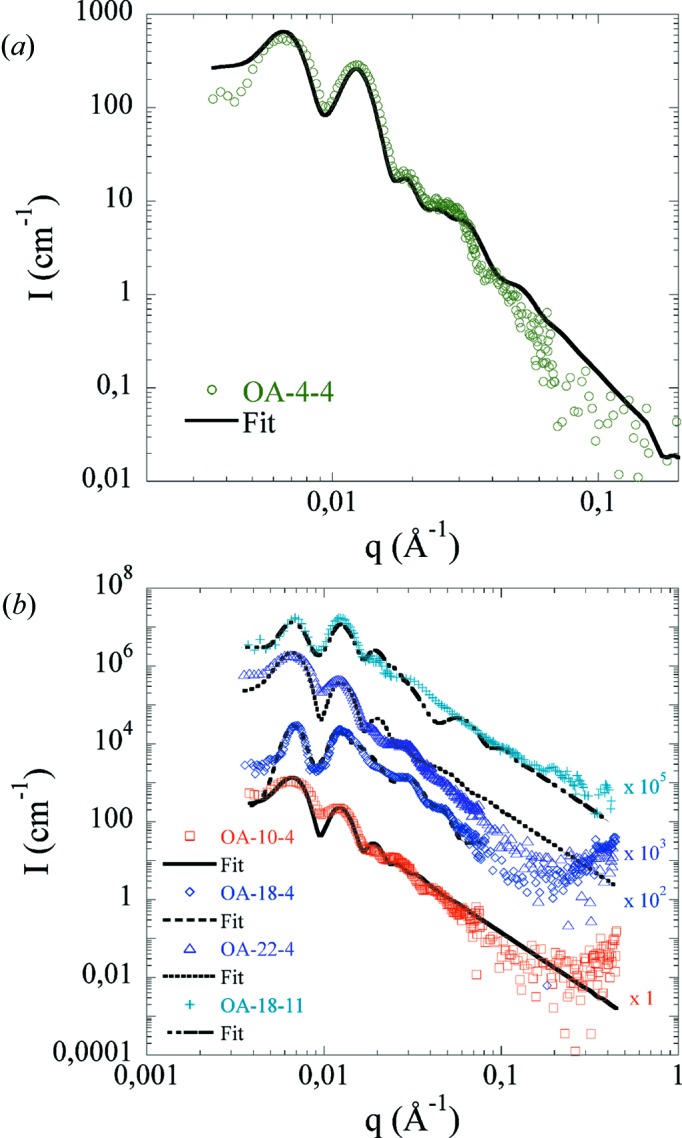
(*a*) SANS scattering intensity of OA-4-4 in 73.2% D_2_O. The continuous black line corresponds to the best fit. (*b*) SANS scattering intensity of OA-10-4 (red squares), OA-18-4 (blue diamonds), OA-22-4 (purple triangles) and OA-18-11 (light-blue crosses) in 73.2% D_2_O. The lines correspond to the best fits. The curves have been shifted for clarity by the factor given on the right.

Fig. 6 ▸(*a*) shows the scattering intensity of OA-4-4 and the corresponding fit obtained by applying our strategy. The parameters derived from the fit are presented in Table 4 ▸. The asterisks (*) indicate parameters that are fixed during the SANS fitting. The relative thickness distribution σ_t_ has also been fixed to an arbitrary value of 0.1 (corresponding to a reasonable relative polydispersity of 10%) because of the low sensitivity of the fit to this parameter.

It can be seen that the core/shell model nicely reproduces the experimental data, validating our fitting strategy. Moreover, the intensity cannot be modeled without a shell, confirming the existence of a contaminated layer. For OA-4-4, we find an *R*_p_ of 31.5 nm (close to the SEM value, around 10% lower) and a shell thickness of 30.4 nm with an SLD (4.58 × 10^−6^ Å^−2^) larger than the bulk SLD (4.47 × 10^−6^ Å^−2^). Fig. 6 ▸(*b*) shows *I*(*q*) of the other AAOs with the best corresponding fits. The curves have been shifted for clarity. For all AAOs the radius found by SANS is close to that determined by SEM. A shell is always necessary and its extent ranges from 10.0 to 30.0 nm, consistent with previous studies (Ono & Masuko, 1992 ▸; Han *et al.*, 2013 ▸). Note that for OA-18-11 the shell thickness (15 nm) is consistent with the value estimated by the dissolution experiment (Fig. 3 ▸). This extent, as well as the shell and bulk SLDs, does not seem to be correlated to the current density, and the different values obtained for the bulk SLD are in the range of 4.4–4.65 × 10^−6^ Å^−2^, in good agreement with recent neutron reflectivity measurements (Christoulaki *et al.*, 2019 ▸).

Then we can use the EDX data to determine the average density of the material for OA-18-11. From the SANS fit, the shell and bulk volume fraction can be determined and an average SLD of 4.55 × 10^−6^ Å^−2^ is calculated. Assuming that the material has an average chemical formula of Al_2_O_3_(C_2_O_4_)_*x*_ (with *x* = C/Al = 0.106; see Fig. 3 ▸), we calculate an average density of 2.976 g cm^−3^, consistent with a previous density determination (Abad *et al.*, 2016 ▸).

All of the AAO samples show small SLD differences between the shell and the bulk, which actually have an important effect on the scattering intensity. The SLD depends on the composition of the material as
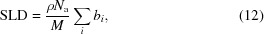
with ρ the density, *N*_a_ the Avogadro number, *M* the molecular weight and *b_i_* the scattering length of atom *i*. A difference in SLD can be explained by a difference in composition or by a difference in the density of the materials. Here, the larger shell SLD can be related to the incorporation of C and O elements (through oxalates) having large *b_i_*, thus increasing the shell SLD (Engel *et al.*, 2009 ▸). Note that the incorporation of OH would have decreased the SLD since H has a negative *b_i_*.

**Figure 7:**
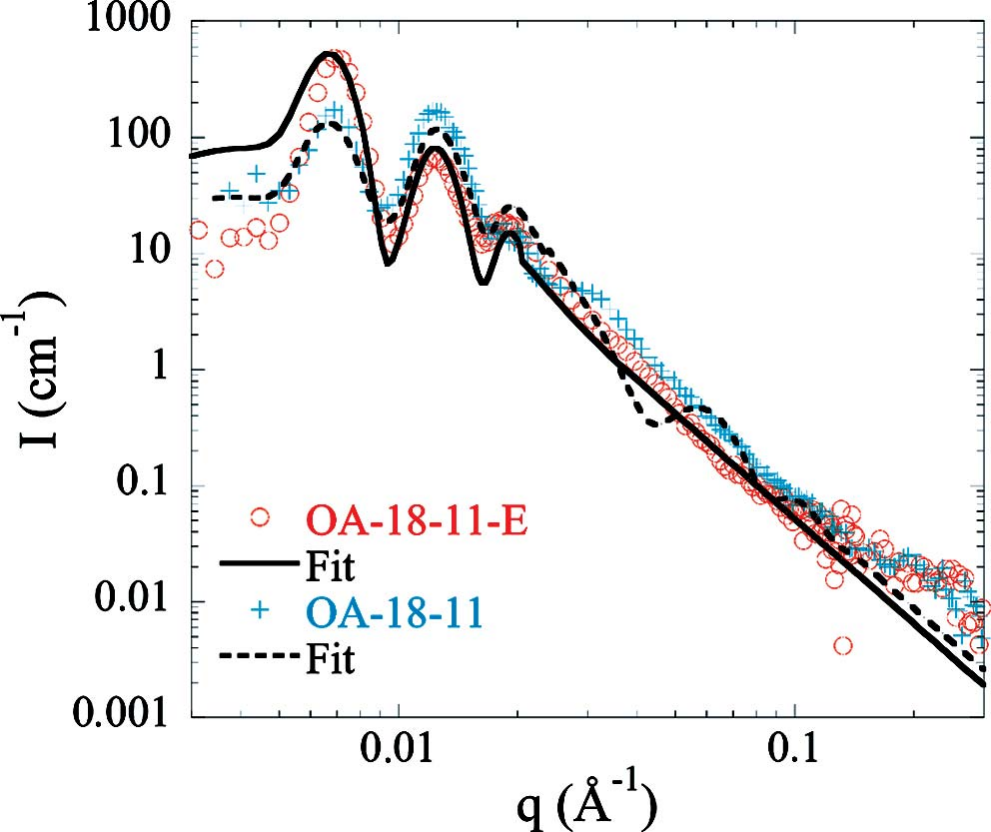
SANS scattering intensity of OA-18-11-E in 73.2% D_2_O (orange circles). The continuous black line corresponds to the best fit. OA-18-11 SANS *I*(*q*) with the fit is also represented for comparison.

To further confirm the existence of the layer and validate its efficient determination through SANS fitting, an AAO equivalent to OA-18-11 was etched for 50 min in phosphoric acid (5 wt% at 30°C). This chemical etching enlarges the pore diameter (*D*_p_ = 89.1 nm, σ_p_ = 0.04, as observed in a SEM image; see Fig. S3) and dissolves the contaminated layer (as described previously in Fig. 3 ▸). The corresponding SANS of this etched sample (OA-18-11-E) is shown in Fig. 7 ▸ with the best fit. The as-prepared AAO (OA-18-11) is also plotted with the fit for comparison. While OA-18-11 needed a shell, the etched sample can be fitted without (see Table 4 ▸). In that case the only fitting parameter is the bulk SLD: we find a value of 4.44 × 10^−6^ Å^−2^, close to the bulk value before etching (*i.e.* 4.50 × 10^−6^ Å^−2^). Using C/Al = 0.0467 (see Fig. 3 ▸ at 50 min) we find a bulk density of 3 g cm^−3^, consistent with a higher-purity alumina region. Finally, from the SANS analysis, we can extract quantitative information about the composition of AAOs.

## 4. Conclusions   

In this paper small-angle neutron scattering measurements coupled with scanning electron microscopy have been performed to gain understanding of the structure and composition of nanoporous alumina membranes. A strategy has been defined to measure AAOs in optimal conditions to avoid multiple scattering effects, and a core/shell cylinder fitting model was found to be appropriate to describe these systems. The existence of a layer contaminated by electrolyte anions (here oxalates) has been confirmed and quantified. We also found that the length in the fitting model is a critical parameter and corresponds to the longitudinal correlation length. Its determination is thus required to fit SANS data in absolute units. Finally, this original approach, based on a detailed and complete description of the SANS data, can be applied to AAOs prepared in different electrolytes (sulfuric or phosphoric acids) and extended to a variety of nanoporous media and will allow the further investigation of condensed matter under confinement.

## Supplementary Material

Description of the AAO alignment, SEM images, EDX data. DOI: 10.1107/S160057671900726X/vg5113sup1.pdf

## Figures and Tables

**Table 1:** Experimental conditions used during the different anodizations The electrolyte was 0.3 *M* oxalic acid (OA) and the voltage was fixed at 40 V for all our experiments.

Names	Temperature (°C)	Time (h)	*j* (mA cm^−2^)	*L*_p_ (µm)†
OA-4-4	4	4	1.90	23
OA-10-4	10	4	2.96	36
OA-18-4	18	4	4.60	58
OA-22-4	22	4	6.90	84
OA-18-11	18	11 h 25 min	4.40	171

† Total AAO thicknesses determined by SEM.

**Table 2:** Characteristic sizes of AAOs obtained by SEM analysis

Name	OA-4-4	OA-10-4	OA-18-4	OA-22-4	OA-18-11
*D*_p_ (nm)	35	33	42	45	53
σ_p_	0.16	0.08	0.09	0.05	0.14
Pore density (10^10^ cm^−2^)	1.1	1.1	1.0	1.0	1.0
Porosity (*P*) (%)	11	9	14	16	23
*L*_p_ (µm)	23	36	58	84	171
*D*_int_ (nm)	103.4	102.6	106.4	103.4	104.5
σ_a_	0.076	0.072	0.063	0.059	0.060
*c*_L_	6.90	6.19	6.48	6.57	6.20
δ (× 10^−4^ Å^−1^)	9.5	8.4	8.2	8.5	9.5
Domain size *D* (nm)	661	748	766	698	661

**Table 3:** Summary of all the parameters present in the model coming from the structure factor *S*(*q*) or the form factor 〈*F*(*q*)^2^〉 Parameters that were fixed during the SANS analysis are marked with an asterisk (*).

Parameters		*F*(*q*) or *S*(*q*)
*D_int_	Interpore distance	*S*(*q*)
*σ_*a*_	Relative interpore distance distribution	*S*(*q*)
*δ/*D*	Peak width/domain size	*S*(*q*)
*m_hk_	Peak multiplicity	*S*(*q*)
*c_L_	Correction factor for Porod invariant	*S*(*q*)
ϕ_s_ = *n* 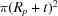	Surface porosity	*F*(*q*)
*ρ_solvent_	Solvent scattering length density	*F*(*q*)
ρ_shell_	Shell scattering length density	*F*(*q*)
ρ_bulk_	Bulk scattering length density	*F*(*q*)
*R*_p_/*σ_p_	Pore radius/pore standard deviation	*F*(*q*)
*t*/*σ_t_	Shell thickness/shell standard deviation	*F*(*q*)
*L_p_	Cylinder length	*F*(*q*)

**Table 4:** Parameters obtained by the fitting of SANS data in Fig. 6 ▸ Parameters that were fixed during the SANS fitting are marked with an asterisk (*).

Name	OA-4-4	OA-10-4	OA-18-4	OA-22-4	OA-18-11	OA-18-11-E
*D_int_ (nm)†	100.0	102.6	106.4	103.4	100.0	100.0
* 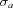	0.076	0.072	0.063	0.059	0.06	0.06
*δ (× 10^−4^ Å^−1^)	9.5	8.4	8.2	8.5	9.5	9.5
*m_hk_	3	3	3	3	3	3
*c*_L_	10	10	10	10	10	10
*ϕ_s_	0.73	0.59	0.24	0.54	0.56	0.66
*ρ_solv_ (10^−6^ Å^−2^)‡	4.52	4.52	4.52	4.52	4.52	4.52
ρ_shell_ (10^−6^ Å^−2^)‡	4.58	4.54	4.71	4.55	4.58	–
ρ_bulk_ (10^−6^ Å^−2^)‡	4.46	4.40	4.65	4.39	4.50	4.44
*R*_p_ (nm)	15.6	16.6	17.4	22.7	26.7	44.5
*σ_p_	0.16	0.08	0.09	0.05	0.14	0.04
*t* (nm)	30.0	27.0	10.0	18.6	15.0	0
*σ_t_	0.10	0.10	0.10	0.10	0.10	–
*L_z_ (µm)	1.49	1.42	1.74	1.40	1.33	1.33

† For OA-4-4 and OA-18-11, *D*_int_ was a fitting parameter.

‡ The errors of the fit are below 0.01%. The error of the solvent SLD is around 0.2%, corresponding to an SLD variation of ±0.01 × 10^−6^ Å^−2^. Thus all the SLD values are given with two digits.
