# Supplementary material for: A novel methodology to study nanoporous alumina by small-angle neutron scattering
Source: J Appl Crystallogr. 2019 Jun 28;52(Pt 4):745–54. doi: 10.1107/S160057671900726X (PMC6662990; doi:10.1107/S160057671900726X)
Supplement: Supplementary file 1 [file j-52-00745-sup1.pdf]

Supplementary Information

A novel methodology to study nanoporous alumina by small-angle neutron scattering

Anastasia Christoulaki[1], Alexis Chennevière[2], Isabelle Grillo[3], Lionel Porcar[3], Emmanuelle Dubois[1] and Nicolas Jouault[1*]

[1]Sorbonne Université, CNRS, Laboratoire Physicochimie des Electrolytes et des Nanosystèmes InterfaciauX, PHENIX, F-75005 Paris, France

[2]Laboratoire Léon Brillouin, LLB, CEA Saclay, 91191 Gif-Sur-Yvette, France

[3]Institut Laue Langevin (ILL), DS/LSS 6 rue Jules Horowitz, 38042 Grenoble Cedex 9, France

Corresponding author:

(NJ): nicolas.jouault@sorbonne-universite.fr

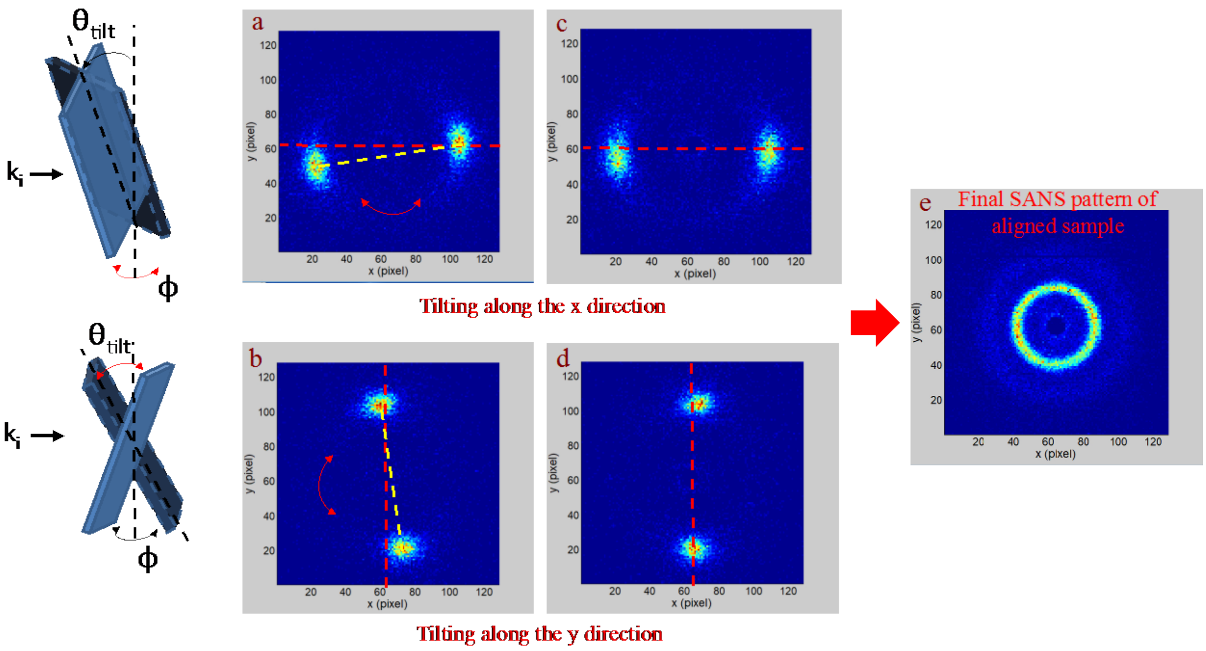

Figure S1. Schematic description of the procedure for AAO alignment along the neutron beam (see main manuscript for details). On the left: schematic representation of the tilting of macroscopic sample. On the right are shown the SANS scattering patterns. The alignment consists in tilting in one direction the sample (X or Y direction), which creates spots on the scattering patterns (a and b) and to slowly move the goniometers in the other direction to align these spots in the X or Y directions (c and d). At the end of this procedure, goniometers are set in their aligned positions and the final SANS pattern is isotropic (e). Any misalignment will create anisotropy in the scattering pattern.

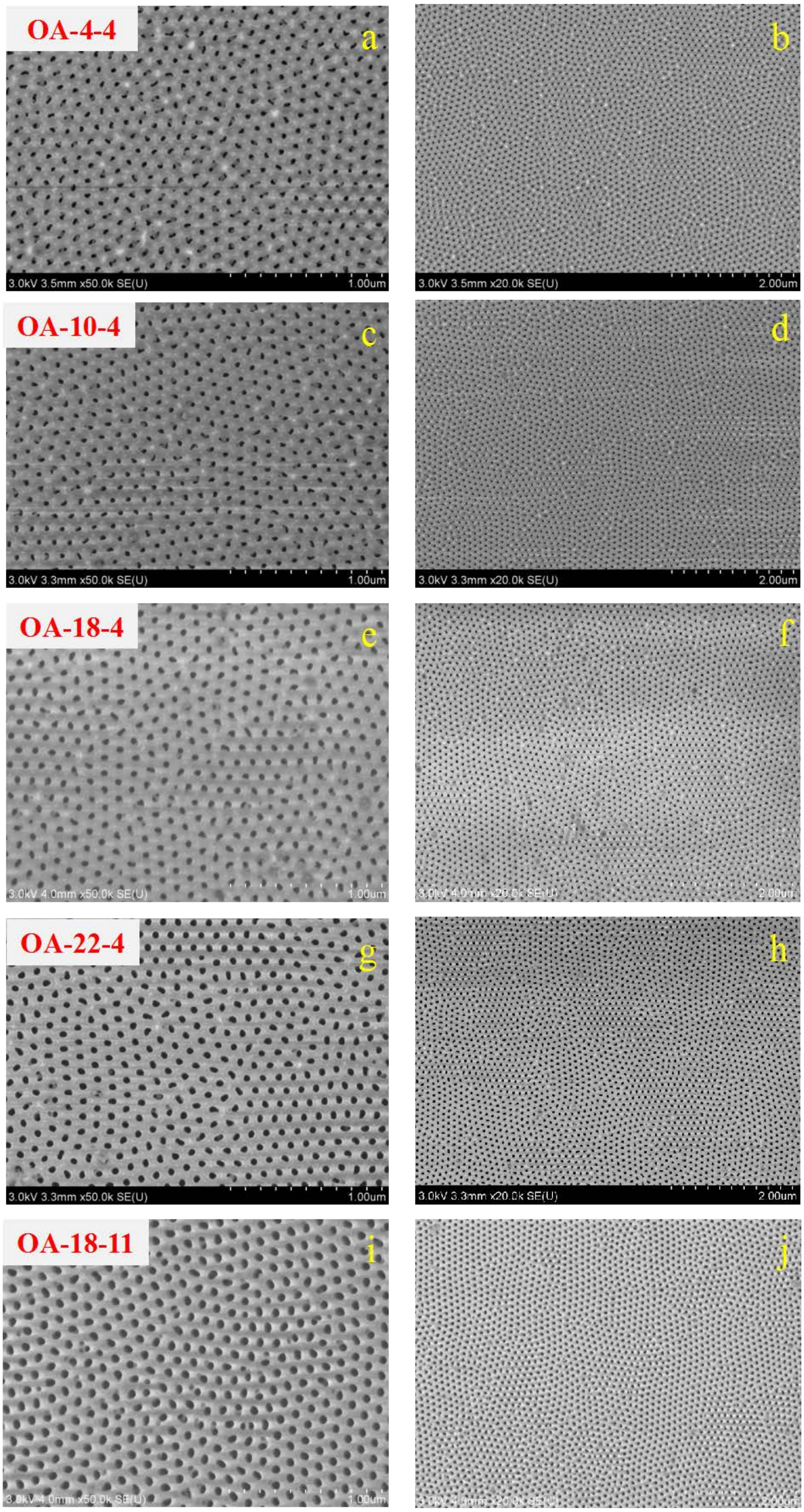

Figure S2. SEM images of (a,b) OA-4-4, (c,d) OA-10-4, (e,f) OA-18-4, (g,h) OA-22-4 and (I, j) OA-18-11 at high (left) and low (right) magnification.

Etching time (min)	Atomic % Al	Atomic % O	Atomic % C	Atomic % P	C/Al
0	32.9	60.7	3.5	2.9	0.106
30	37.2	58.9	2.1	1.7	0.056
50	36.4	59.9	1.7	2.1	0.047

Table S1. EDX elemental surface analysis of OA-18-11 after different etching time in 5 %wt phosphoric acid at 30°C.

**Name**	**OA-4-4**	**OA-10-4**	**OA-18-4**	**OA-22-4**	**OA-18-11**
**Instrument**	D11	D11	D11	D11	PAXY
**Collimation length (m)**	16.5	16.5	40.5	16.5	4.75
**Wavelength (Å)**	6	6	6	6	15
**ΔΘ (°)**	0.00052	0.00052	0.00021	0.00052	0.0012
**L_T (nm)**	1162	1162	2850	1162	1229
**δ (x 10^{-4} $Å^{-1}$)**	5.4	5.4	2.2	5.4	5.1

Table S2. Experimental collimations used to define the divergence of the direct beam and the transverse coherent length L_T.

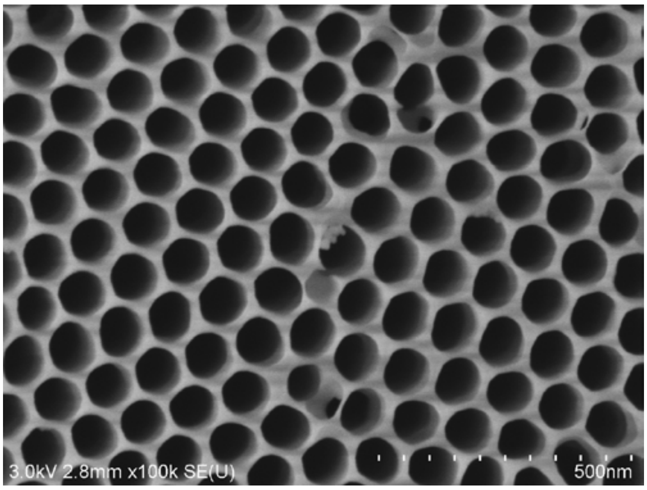

Figure S3. SEM image of OA-18-11 etched 50 min in phosphoric acid.
